# Spatial Dispersion in Hypercrystal Distributed Feedback Lasing

**DOI:** 10.3390/ma15103482

**Published:** 2022-05-12

**Authors:** Bartosz Janaszek, Paweł Szczepański

**Affiliations:** 1 Institute of Microelectronics and Optoelectronics, Warsaw University of Technology, Koszykowa 75, 00-665 Warsaw, Poland; pawel.szczepanski@pw.edu.pl; 2 National Institute of Telecommunications—The State Research Institute, 1 Szachowa Str., 04-894 Warsaw, Poland

**Keywords:** photonic hypercrystals, spatial dispersion, hyperbolic metamaterials, DFB laser

## Abstract

This work is a first approach to investigate the role of spatial dispersion in photonic hypercrystals (PHCs). The scope of the presented analysis is focused on exploiting nonlocality, which can be controlled by appropriate design of the structure, to obtain new light generation effects in a distributed feedback (DFB) laser based on PHC, which are not observable under weak spatial dispersion. Here, we use effective medium approximation and our original model of threshold laser generation based on anisotropic transfer matrix method. To unequivocally identify nonlocal generation phenomena, the scope of our analysis includes comparison between local and nonlocal threshold generation spectra, which may be obtained for different geometries of PHC structure. In particular, we have presented that, in the presence of strong spatial dispersion, it is possible to obtain spectrally shifted Bragg wavelengths of TE- and TM-polarization spectra, lowered generation threshold levels for both light polarizations, generation of light of selected light polarization (TE or TM), or simultaneous generation of TE- and TM-polarized waves at different frequencies with controllable spectral separation, instead of single mode operation anticipated with local approach.

## 1. Introduction

Recently, the influence of spatial dispersion (nonlocality), which, in the optical domain optics, can be understood as wavevector-dependence of electric permittivity, on properties of nanostructures has gathered widespread attention from scientists around the world [1,2,3,4,5,6]. Recent studies revealed that nonlocality plays a significant role in shaping properties of nanostructural metamaterials, such as wire media [1], metallic nano-particle arrays [2], or split ring resonators [3]. The particular importance of considering spatial dispersion has been proven in the development of hyperlenses [4] and scatter-free detectors [5]. Moreover, it has been also demonstrated that, by accounting for nonlocality, it is possible to predict the existence of parasitic signal channel arising from the presence of additional waveguide mode [6].

Due to the fact that strength of nonlocality in a nanostructure may be controlled by, e.g., appropriate geometrization [7,8] or inducing asymmetry [9,10], the more recent studies are concentrated on utilization of spatial dispersion to obtain new functionalities which cannot be predicted with the help of local approximation. In particular, it has been demonstrated that nonlocality in metamaterials may lead to enhancement of nonlinear response [11] or spontaneous emission [12]. Moreover, nonlocal response of hyperbolic metamaterials may result in new effects, such nonlocal quantum gain of plasmons [13], blueshift of intramolecular charge transfer emission [14], selective spatial filtering [15], or nonmagnetic optical isolation [16,17]. Furthermore, strong spatial dispersion enables us to obtain new degrees of freedom in controlling direction [18] and threshold of Cherenkov radiation [19].

Despite increasing number of studies in the field of nonlocal metamaterials, the effect of spatial dispersion on optical properties of photonic hypercrystals has not been yet discussed. Photonic hypercrystals (PHCs) are a novel class of metamaterials combining properties of photonic crystals and hyperbolic media [20] and revealing interesting properties, such as difractionless imaging [21], optical bistability [22], cloaking [23], or tunable broadband unidirectional absorption [24]. Recently, it has also been demonstrated that tunable lasing with voltage-controllable single mode generation is also possible in this class of structure [25].

In this work, we study the role of spatial dispersion in shaping properties of PHC structures, which has not been yet investigated. The scope of investigation is concentrated on light generation in distributed feedback (DFB) lasers based on photonic hypercrystals in the presence of spatial dispersion. Following our previous research [8,26], the presented analysis includes PHC structures based on hyperbolic media of various geometries, which correspond to different nonlocal effects. To unveil these nonlocal generation effects, we have demonstrated the difference between threshold generation spectra predicted with the help of local and nonlocal approach. In particular, we have demonstrated that by use of a nonlocal approach it is possible to correctly anticipate generation properties arising in the presence of the spatial dispersion, such as spectrally shifted Bragg wavelengths of TE- and TM-polarized waves or lowered generation threshold levels for both light polarizations. Moreover, it has been demonstrated that nonlocality in PHC laser may be utilized to obtain versatile generation properties, such as generation of light of selected (TE or TM) light polarization or simultaneous generation of TE- and TM-polarized waves at different frequencies, instead of single mode generation predicted with the help of local approximation.

## 2. Theory

In this section we describe the theoretical framework which has been used to describe propagation and light generation in the considered photonic hypercrystal laser. The first section is focused on effective description of hyperbolic medium via use of local and nonlocal effective medium theory (EMT). The second section contains a description of transfer matrix method (TMM) approach for anisotropic media, which allows us to formulate threshold conditions for generation of a planar PHC laser. It is worth underlining that the chosen analytical methods have been widely recognized as a reliable description of light propagation in planar metamaterials [27,28,29,30,31].

### 2.1. Local and Nonlocal Effective Medium Theory

According to the basic principles of effective medium theory [32], an inhomogeneous nanostructure composed of isotropic materials may be described as a homogeneous anisotropic medium with effective permittivity tensor with diagonal components $\overset{=}{\varepsilon }=diag\left(\left[\begin{array}{ccc} \varepsilon_{xx}^{loc}, & \varepsilon_{yy}^{loc}, & \varepsilon_{zz}^{loc} \end{array}\right]\right)$.

**Figure 1:**
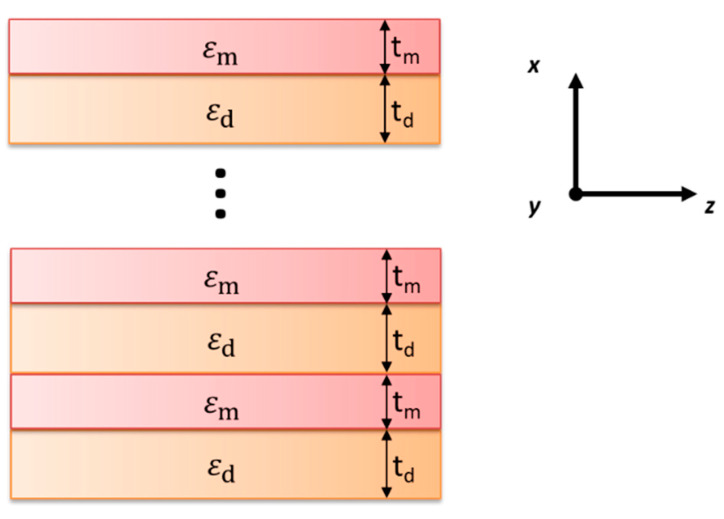
Schematic illustration of hyperbolic metamaterial.

In particular, a planar nanostructure, see Figure 1, composed of a periodical arrangement of two different materials, i.e., plasmonic and dielectric, can be effectively described as uniaxially anisotropic with the permittivity tensor of components in the following form [30]

(1) $$\varepsilon_{zz}^{loc}=\varepsilon_{yy}^{loc}=\varepsilon_{∥}=\frac{t_{d}\varepsilon_{d}+t_{m}\varepsilon_{m}}{t_{d}+t_{m}},$$

(2) $$\varepsilon_{xx}^{loc}=\varepsilon_{⊥}=\frac{\varepsilon_{d}\varepsilon_{m}\left(t_{d}+t_{m}\right)}{t_{d}\varepsilon_{m}+t_{m}\varepsilon_{d}},$$

where $\varepsilon_{m}$, $\varepsilon_{d}$, as well as $t_{m}$, $t_{d}$ are, respectively, relative permittivity and thickness of plasmonic and dielectric layers constituting the structure. It is worth noting that this approximation is valid if local condition is fulfilled, i.e., the dimensions of the unit cell are subwavelength, i.e., $\frac{t_{m}+t_{d}}{\lambda }\to 0$, where λ is wavelength of considered radiation.

However, the local approximation fails to account for the influence of effects related to the presence of spatial dispersion, which may be induced by violation of the local conditions or existence of high-wavevector states [33,34]. To properly describe the influence of the spatial dispersion, a proper adjustment of the local EMT approach is required. In this work, we use the nonlocal EMT formalism derived by Chern [35], which enables us to predict properties of a planar metamaterial composed of plasmonic and dielectric when the local condition is violated, i.e., the size of the unit cell is no longer negligible in comparison with the wavelength considered $\;\left(t_{m}+t_{d}\right)/\lambda_{0}↛$ 0.

In general, a planar inhomogeneous nanostructure, see Figure 1, composed of a periodical arrangement of two different materials, i.e., plasmonic and dielectric, may be described with dispersion relations for TE- and TM-polarized waves, respectively [35,36]:

(3) $$cos\left(k_{z}t\right)=cos\left(q_{1}t_{d}\right)cos\left(q_{2}t_{m}\right)-\frac{1}{2}\left(\frac{q_{1}}{q_{2}}+\frac{q_{2}}{q_{1}}\right)sin\left(q_{1}t_{d}\right)sin\left(q_{2}t_{m}\right),$$

(4) $$cos\left(k_{z}t\right)=cos\left(q_{1}t_{d}\right)cos\left(q_{2}t_{m}\right)-\frac{1}{2}\left(\frac{\varepsilon_{m}q_{1}}{\varepsilon_{d}q_{2}}+\frac{\varepsilon_{d}q_{2}}{\varepsilon_{m}q_{1}}\right)sin\left(q_{1}t_{d}\right)sin\left(q_{2}t_{m}\right),$$

where $q_{1}=\sqrt{\varepsilon_{d}k_{0}^{2}-k_{x}^{2}}$, $q_{2}=\sqrt{\varepsilon_{m}k_{0}^{2}-k_{x}^{2}}$ while $t=t_{d}+t_{m}$. On the other hand, dispersion relations for TE and TM polarized waves in a homogenous anisotropic medium may be described as follows:

(5) $$k_{x}^{2}+k_{z}^{2}=\varepsilon_{yy}^{eff}k_{0}^{2},$$

(6) $$\frac{k_{x}^{2}}{\varepsilon_{zz}^{eff}}+\frac{k_{z}^{2}}{\varepsilon_{xx}^{eff}}=k_{0}^{2}.$$

According to approach developed by Chern [35], spatial dispersion may be accounted by expanding Equations (3) and (4) into Taylor series for wavevector components $k_{x},k_{z}$. Then, by neglecting terms higher than the fourth order, it is possible to transform the considered equation into the algebraic form of dispersion relation for an anisotropic homogeneous medium, see Equations (5) and (6), which allows us to obtain analytical formulas for permittivity tensor components:

(7) $$\varepsilon_{xx}^{nloc}=\frac{\varepsilon_{⊥}-\frac{\alpha }{12}k_{0}^{2}t^{2}}{1+\frac{\varepsilon_{⊥}}{\varepsilon_{\left|\right|}}\left(\frac{\beta }{12}k_{z}^{2}t^{2}-\frac{\gamma }{6}k_{0}^{2}t^{2}\right)},$$

(8) $$\varepsilon_{yy}^{nloc}=\varepsilon_{\left|\right|}\left(1+\frac{1}{6}k_{z}^{2}t^{2}\right)+\frac{t^{2}}{12k_{0}^{2}}\left(k_{x}^{4}-k_{z}^{4}\right)-\frac{\alpha }{12}k_{0}^{2}t^{2},$$

(9) $$\varepsilon_{zz}^{nloc}=\frac{\varepsilon_{\left|\right|}-\frac{\alpha }{12}k_{0}^{2}t^{2}}{1-\frac{1}{12}k_{x}^{2}t^{2}}$$

where $k_{x},k_{z}$ are components of wavevector of the wave inside medium and $k_{0}$ is freespace wavevector, while nonlocal coefficients α, β*,* and γ can be formulated in the following form:

(10) $$\alpha =\left[f_{m}^{2}\varepsilon_{m}\left(\omega \right)+\left(1-f_{m}^{2}\right)\varepsilon_{d}\right]\left[\left(1-f_{d}^{2}\right)\varepsilon_{m}+f_{d}^{2}\varepsilon_{d}\right],$$

(11) $$\beta =\frac{1}{\varepsilon_{m}\varepsilon_{d}\left(\omega \right)}\left[\left(1-2f_{m}f_{d}\right)\varepsilon_{m}+2f_{m}f_{d}\varepsilon_{d}\right]\left[2f_{m}f_{m}\varepsilon_{m}+\left(1-2f_{m}f_{d}\right)\varepsilon_{d}\right],$$

(12) $$\gamma =\frac{1}{\varepsilon_{m}\varepsilon_{d}}\left[f_{m}^{3}f_{d}\varepsilon_{m}^{3}+f_{m}\left(1-2f_{m}^{2}f_{d}+f_{d}^{3}\right)\varepsilon_{m}^{2}\varepsilon_{d}+f_{m}\left(1-f_{m}f_{d}^{2}+f_{m}^{3}\right)\varepsilon_{m}\varepsilon_{d}^{2}+f_{m}f_{d}^{3}\varepsilon_{d}^{3}\right]$$

where $f_{m}=t_{m}/\left(t_{d}+t_{m}\right)$ and $f_{d}=t_{d}/\left(t_{d}+t_{m}\right)$ are, respectively, the filling ratios of plasmonic and dielectric layers constituting the multilayer hyperbolic medium. Note that EMT formalism is valid for structure constructed of at least 6 unit cells [37]. It is worth underlining that, due to accounting for wavevector components up to the fourth order in the dispersion relation of periodical medium, see Equations (3) and (4), the presented EMT formalism allows us to perform “full” homogenization of the structure, including material properties, wavevector, media interfaces, etc., with high accuracy up to a few percent error [35]. In particular, under considered conditions, propagating waves may be considered to reveal constant phase along the homogenized HMM medium. It is also worth underlining that the employed nonlocal EMT approach [35] takes into account influence of coupling between surface plasmons propagating at each metal/dielectric interface and bulk modes propagating in dielectric layers, which is of key importance in shaping properties of planar metamaterial [38].

### 2.2. Transfer Matrix Method Approach: Determination of Threshold Condition

The main principle behind transfer matrix method is an assumption that a planar layer can be described with characteristic matrix Ω [39]

(13) $$\Omega =\left[\begin{array}{cccc} 0 & 0 & \frac{1}{\varepsilon_{z}}\bar{k_{x}}\bar{k_{y}} & \mu_{y}-\frac{{\bar{k_{x}}}^{2}}{\varepsilon_{z}}\\ 0 & 0 & \frac{{\bar{k_{y}}}^{2}}{\varepsilon_{z}}-\mu_{x} & -\frac{1}{\varepsilon_{z}}\bar{k_{x}}\bar{k_{y}}\\ \frac{1}{\mu_{z}}\bar{k_{x}}\bar{k_{y}} & \varepsilon_{y}-\frac{{\bar{k_{x}}}^{2}}{\mu_{z}} & 0 & 0\\ \frac{{\bar{k_{y}}}^{2}}{\mu_{z}}-\varepsilon_{x} & -\frac{1}{\mu_{z}}\bar{k_{x}}\bar{k_{y}} & 0 & 0 \end{array}\right],$$

where $\varepsilon_{x},\varepsilon_{y},\varepsilon_{z}$ as well as $\mu_{x},\mu_{y},\mu_{z}$ are components of permittivity and permeability diagonal tensors of the considered anisotropic layer and $\bar{k_{x,y,z}}=k_{x,y,z}/k_{0}\;$ are components of normalized wavevector. It is worth underlining that absorption and gain of material is accounted with imaginary part of permittivity. The characteristic matrix Ω is further used to solve the problem of light propagation through the considered layer, which may be represented in the form of matrix equation [15]

(14) $$\frac{\partial \psi }{\partial z^{\prime }}-\Omega \psi =0,$$

where

(15) $$\psi =\left[\begin{array}{c} E_{x}\left(z^{\prime }\right)\\ E_{y}\left(z^{\prime }\right)\\ \bar{H_{x}}\left(z^{\prime }\right)\\ \bar{H_{y}}\left(z^{\prime }\right) \end{array}\right].$$

It is noteworthy that normalization of magnetic field ${\bar{H}}_{x,y}=-j\sqrt{\mu_{0}/\varepsilon_{0}}H_{x,y}$ and position $z^{\prime }=z/k_{0}$ is employed. The solution of Equation (14) may be assumed as $\psi \left(z^{\prime }\right)=e^{\Omega z^{\prime }}\psi \left(0\right).\;$ However, due to matrix form of the exponential function argument, it may reformulate to a subsidiary eigenvalue problem $\psi \left(z^{\prime }\right)=We^{\Lambda z^{\prime }}c$*,* where $c=W^{-1}\psi \left(0\right)$ is vector of field amplitudes and W and Λ are eigen vector and eigenvalue matrices of the characteristic matrix Ω of the layer, which may be calculated via established numerical methods [40]. It is worth noting that the vector of field amplitudes $c=\left[\begin{array}{cccc} c_{TE}^{+} & c_{TE}^{-} & c_{TM}^{+} & c_{TM}^{-} \end{array}\right]$ describes all modes, i.e., backward and forward propagating waves with TE (with field components $E_{y},H_{x},H_{z}$) and TM (with field components $H_{y},E_{x},E_{z}$) polarizations, that exists in the medium. More detailed description of presented approach may be found in our previous research [15,16,25]. Finally, by assuming appropriate conditions for adjacent layers/media, it is possible to formulate a relationship between amplitudes at the input *c_in_* and output side *c_out_* of a single layer

(16) $$c_{out}=T\cdot c_{in},$$

where $T=W_{out}^{-1}We^{\Lambda k_{0}L}$ is the transfer matrix of the layer, L is the thickness of the considered layer, while $W_{out}$ is eigenvector matrix of output layer/medium. With the help of such an approach, it is possible to represent the complete multilayer structure as a multiplication of transfer matrices of the constituent layers

(17) $$T_{global}=W_{N+1\left(out\right)}^{-1}W_{N}e^{\lambda_{N}k_{0}L_{N}}\cdot W_{N}^{-1}W_{N-1}e^{\lambda_{N-1}k_{0}L_{N-1}}\cdot ...\cdot W_{2}^{-1}W_{1}e^{\lambda_{1}k_{0}L_{1}}\cdot W_{1}^{-1}W_{0\left(in\right)},$$

where $W_{0\left(in\right)}$ and $W_{N+1\left(out\right)}$ are eigenvector matrices of media surrounding the structure. Now, we have a model of light propagation through the whole multilayer structure.

**Figure 2:**
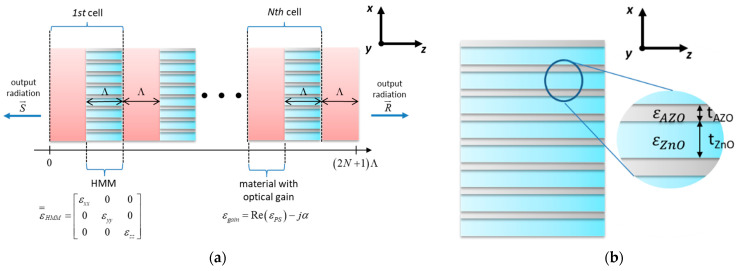
Scheme of a DFB laser based on photonic hypercrystal (**a**) and a hyperbolic metamaterial based on AZO and ZnO (**b**).

Along with Kogelnik’s model [40], the laser action in our analysis is understood as a result of oscillations arising from a reflection at the gain medium/air interface and further energy accumulation via scattering from counter-propagating waves inside the structure. Thus, the laser threshold is considered as nonzero output under no external incident radiation and assumed reflection conditions [25]. By combining Equations (13) and (14), (16) as well as assuming that the structure ends with gain medium on both sides, see Figure 2a, it is possible to obtain an expression for threshold generation in such a structure [40]

(18) $$\left[\begin{array}{c} \tau \\ 0\\ \tau \\ 0 \end{array}\right]=T_{global}\cdot \left[\begin{array}{c} 0\\ -\tau \\ 0\\ -\tau \end{array}\right],$$

where $\tau =2n_{g}/\left(n_{g}+1\right)$ is Fresnel transmission amplitude coefficient for the gain medium-air interfaces. Finding the roots of Equation (18) allows us to determine modal spectra illustrating gain thresholds, i.e., the amount of gain that is required to obtain generation, for TE and TM longitudinal modes of a DFB laser based on photonic hypercrystal. It is worth underlining that, by application of proper boundary conditions, we consider only normal far-field modes of the DFB laser, i.e., waves propagating along z axis with wavevector $\vec{k}=\left[0,0,k_{0}\right]$.

Along with our previous research [25], amplitude gain coefficient, which is used for calculation of generation thresholds, is expressed in the following form:

(19) $$g=4\pi \frac{\sqrt{0.5\left(\left|\varepsilon_{gain}\right|-Im\left(\varepsilon_{gain}\right)\right)}}{\lambda_{0}\left[\mathrm{cm}\right]},$$

where $\varepsilon_{gain}$ is complex permittivity of the gain medium, while the Bragg wavelength of a PHC laser is defined as follows

(20) $$\lambda_{bragg}=\Lambda \cdot \left(\left|n_{HMM}\right|+\left|n_{g}\right|\right),$$

where Λ is the size of the PHC’s unit cell, see Figure 2a, and $n_{HMM}\approx \sqrt{\varepsilon_{yy}}$, $n_{g}\approx \sqrt{\varepsilon_{gain}}$ are refractive indices of hyperbolic and the gain media, respectively.

## 3. Results

In this section, we investigate the influence of spatial dispersion on generation of a DFB laser based on a PHC structure. In the first section, we describe the considered PHC laser and discuss the chosen materials. The second section is focused on analysis of influence of nonlocality on generation properties of PHC laser. The analysis consists of a few chosen cases related to different geometries of hyperbolic medium, and, thus, different strengths of nonlocality. The last section is dedicated to analysis of selected DFB lasers based on photonic hypercrystal with realistic gain material.

### 3.1. Distributed Feedback Laser Based on Hypercrystal

In this work, we investigate threshold laser action in a DFB laser based on photonic hypercrystal (PHC), see Figure 2a. The distributed feedback in the considered structure is realized with periodically arranged layers formed with dielectric material with optical gain and hyperbolic medium (HMM structure), see Figure 2b. In this case, polystyrene polymer (PS) has been chosen as an example of gain dielectric medium with complex permittivity $\varepsilon_{gain}=Re\left(\varepsilon_{PS}\right)-j\alpha$, where α correspond to gain of the medium [41]. To obtain optical gain in such a medium, doping with various dye molecules, e.g., rhodamine or DCM, may be performed [42,43]. On the other hand, we consider the HMM medium to be formed with subsequent layers of zinc oxide (ZnO) and zinc oxide with 2.08% concentration of aluminum (AZO), which can be described with well-established dielectric functions [44,45]. To determine influence of the spatial dispersion on generation properties in such laser, the HMM structure is described with the use of local, i.e., ${\overset{=}{\varepsilon }}_{HMM}=diag\left(\left[\varepsilon_{xy}^{loc},\varepsilon_{xy}^{loc},\varepsilon_{zz}^{loc}\right]\right)$, see Equations (1) and (2), and nonlocal effective medium approximations, i.e., ${\overset{=}{\varepsilon }}_{HMM}=diag\left(\left[\varepsilon_{xx}^{nloc},\varepsilon_{yy}^{nloc},\varepsilon_{zz}^{nloc}\right]\right)$, see Equations (7) and (9).

The considered structure is composed of *N* = 25 basic cells with an additional active polystyrene layer at the end of the structure, which provides symmetrical truncating conditions (air/polystyrene interface) for output amplitudes, see Equation (18). Additionally, parallel orientation of layers constituting the HMM structure with respect to propagation direction ensures invariance of wavevector of waves, i.e., continuity of wavevector parallel to the boundary induce constant phase along the hyperbolic medium, which validates application of EMT approach for calculating propagation. Note that the considered constituent materials were chosen as an example to present more general phenomena arising from effective nonlocality of periodical HMM structure. However, it is still worth underlining that the general form of considered PHC laser may be fabricated via usage of well-established techniques, i.e., (1) deposition of multilayer HMM structure via ALD [46] or magnetron sputtering [47], (2) defining grating of the assumed period of the PHC structure Λ via e.g., electron-beam lithography [48] or phase-mask lithography [49], or (3) deposition of the chosen active material via respective technological process [42,43,50].

### 3.2. Modal Spectra

**Figure 3:**
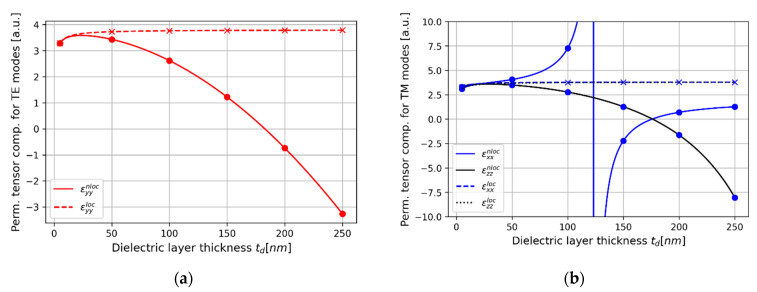
Real parts of local and nonlocal permittivity tensor components for TE- (**a**) and TM-polarized waves (**b**) as a function of dielectric thickness. The cases selected for analysis, i.e., $t_{d}=t_{ZnO}$ = 5 (case 1), 50 (case 2), 100 (case 3), 150 (case 4), 200 (case 5), 250 nm (case 6) have been denoted with dots for nonlocal curves and x-marks for local curves.

Here, we consider a PHC laser, see Figure 2a, based on hyperbolic media composed of various basic cells with $t_{m}$ = *t_AZO_* = 2.5 nm layer of AZO and different ZnO layer thicknesses, i.e., $t_{d}$ = *t_ZnO_* = 5 (case 1), 50 (case 2), 100 (case 3), 150 (case 4), 200 (case 5), 250 nm (case 6). It is noteworthy that each selected dielectric thickness (denoted with red and blue markers for TE- and TM- polarized waves, respectively) corresponds to different dispersion properties for TE- and TM-polarization, see Figure 3a,b. Consistently with our previously presented results [8], the influence of spatial dispersion is sufficiently strong to cause alteration of dielectric, predicted with the local EMT, to other types of dispersion, i.e., epsilon-near-zero (ENZ, $0<\varepsilon_{yy}<1$) or metallic dispersion ($\varepsilon_{yy}<0$) for TE-polarized waves and ENZ ($0<\varepsilon_{xx}<1$), Type I ($\varepsilon_{xx}>0,\varepsilon_{zz}<0$) or Type II hyperbolic dispersion ($\varepsilon_{xx}<0,\varepsilon_{zz}>0$) for TM-polarized waves.

For each considered case, the size of the basic cell of the PHC, i.e., the layer thickness Λ, is calculated based on the local permittivity tensor components of HMM structure with the use of Equations (1) and (2) and assumed Bragg wavelength $\lambda_{bragg}$. = 630 nm. To illustrate influence of nonlocality, for each dielectric thickness, TE- and TM-polarization modal spectra for a structure described with local and nonlocal EMT have been set together in a single figure. It is worth noting that the resulting modal spectra are superposition of two feedback mechanisms that arise in the considered structure, i.e., reflection from the end mirrors (air/polystyrene interfaces) and distributed feedback, provided by periodical arrangement of the PHC structure. Moreover, the distributed feedback is influenced by two factors, i.e., index- and gain/loss modulations across the structure, which are characterized with different generation properties [40,51]. Thus, it can be expected that modal spectra may significantly vary, depending on the dominating feedback mechanism.

#### 3.2.1. Case 1–5 nm Dielectric Layer

**Figure 4:**
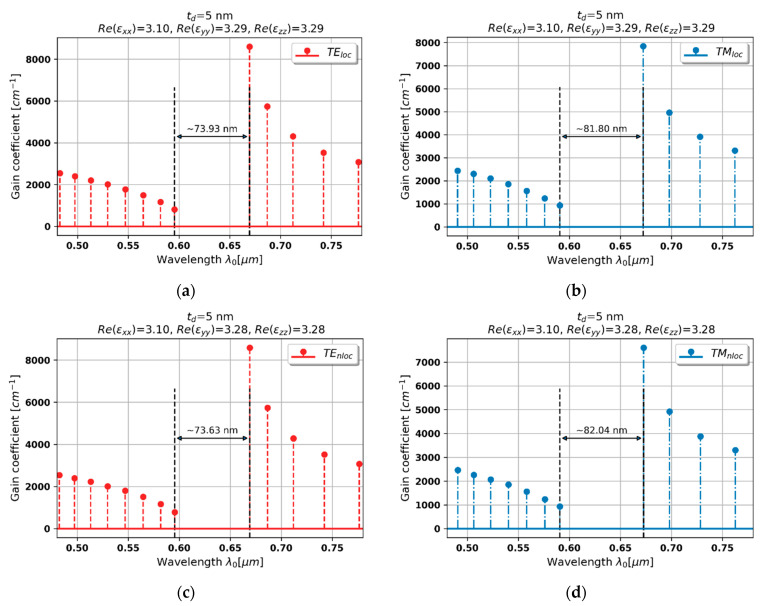
Modal spectra for TE (**a**,**c**) and TM-polarized light (**b**,**d**) for a PHC laser based on hyperbolic medium with $t_{ZnO}$ = 5 nm dielectric layer (black double-headed arrow denotes spectral width of stop band of the spectrum).

In the case of the PHC with HMM based on dielectric layer thickness *t_ZnO_* = 5 nm, due to dielectric character of dispersion, all observed generation spectra are similar to behavior of a conventional DFB laser with small gain/loss- and dominant index-modulation [25,40]. Moreover, it can be observed that generation properties predicted with the local EMT approach are very consistent with the nonlocal response for both light polarizations, compare Figure 4a,b and Figure 4c,d. This can be explained by the fact that locality condition, i.e., t/λ→ 0, where $t=t_{ZnO}+t_{AZO}$, is preserved, and thus, the influence of spatial dispersion is negligible.

It can be also observed that local and nonlocal spectra for TE and TM polarizations are shifted with respect to each other. This phenomenon is caused by the fact that both a local and nonlocal description predicts that waves of different polarization perceive a slightly different medium, i.e., $Re\left(\varepsilon_{yy}\right)=3.28$ for TE polarization and $Re\left(\varepsilon_{xx}\right)=3.10$ for TM-polarized waves, compare Figure 4a,c and Figure 4b,d.

#### 3.2.2. Case 2–50 nm Dielectric Layer

**Figure 5:**
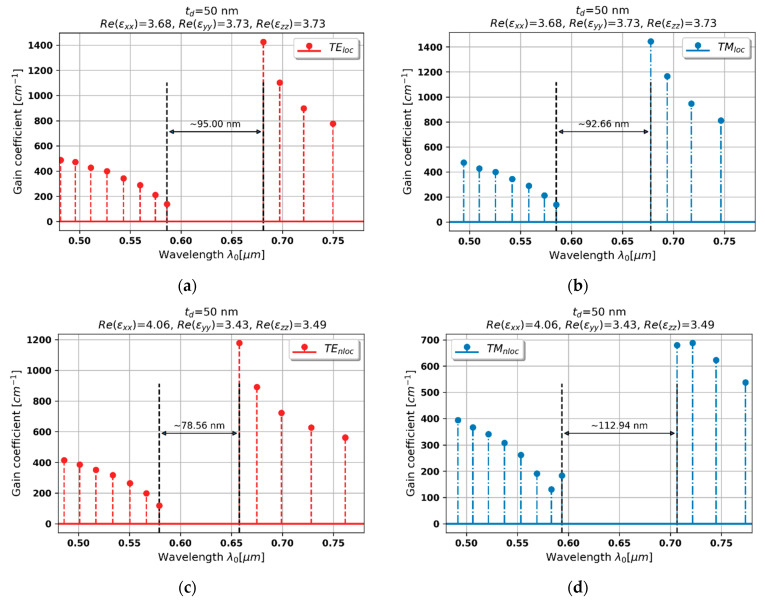
Modal spectra for TE (**a**,**c**) and TM-polarized light (**b**,**d**) for a PHC laser based on hyperbolic medium with $t_{ZnO}$ = 50 nm dielectric layer (black double-headed arrow denotes spectral width of stop band of the spectrum).

Increasing the dielectric thickness leads to violation of the local condition, which consequently leads to stronger spatial dispersion and influences generation properties of the considered laser. In the case of HMM structure based on 50 nm dielectric thickness, the dielectric dispersion for both light polarization is preserved, see Figure 3a,b. However, due to lower permittivity, and thus optical density, the stop band for TE-polarized waves, predicted with the help of nonlocal approach (further referred as nonlocal generation spectrum), is narrower and blueshifted with respect to the generation spectrum based on local EMT description (further referred as local generation spectrum), compare Figure 5a and Figure 5c. Moreover, the gain thresholds predicted with the nonlocal approach (further referred as nonlocal gain thresholds) are noticeably lower than their local counterparts, compare Figure 5a,b and Figure 5c,d.

By accounting the nonlocality, it is also possible to anticipate significant changes in modal generation spectrum for TM-polarized light. This time, the nonlocal approach allows us to predict lower refractive index $n_{TM}\approx \sqrt{\varepsilon_{xx}}$, which leads to a wider and redshifted stop band in comparison with local response. What is more, the generation thresholds for modes of longer wavelengths (right side of the spectrum) become significantly lower when the spatial dispersion is accounted for, compare Figure 5b and Figure 5d.

These phenomena are caused by the fact that waves travelling inside the medium perceive different optical properties, i.e., permittivity, rather than those predicted with the local approach, which significantly affects mechanisms of distributed feedback that arise for each light polarization. It is worth underlining that, due to symmetrical truncation with gain medium, the contribution of Fabry–Perot in shaping feedback mechanism is independent of nonlocal effects and remains unchanged in our analysis.

#### 3.2.3. Case 3–100 nm Dielectric Layer

Constructing the PHC laser based on HMM with dielectric layer 100 nm leads to further changes in generation properties, despite the fact that dispersion is still not altered, i.e., dielectric dispersion is preserved for TE- and TM-polarized waves, see Figure 3a,b.

**Figure 6:**
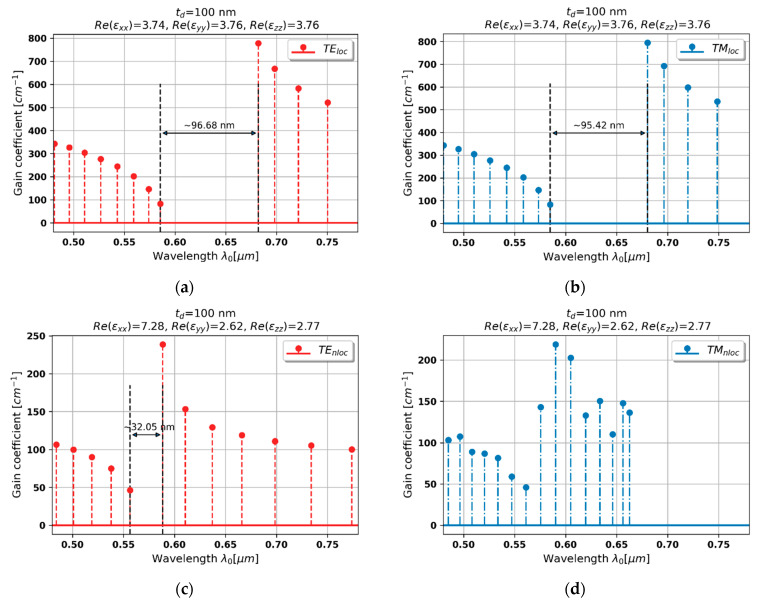
Modal spectra for TE (**a**,**c**) and TM-polarized light (**b**,**d**) for a PHC laser based on hyperbolic medium with $t_{ZnO}$ = 100 nm dielectric layer (black double-headed arrow denotes spectral width of stop band of the spectrum).

By accounting spatial dispersion, it is possible to predict lower optical density for TE-polarized waves propagating in the hyperbolic medium, i.e., lower refractive index $n_{TE}=\sqrt{\varepsilon_{yy}}$. This phenomenon leads to narrowing and blueshifting of the stop band with respect to the previous case, compare Figure 5c and Figure 6a with Figure 6c. It is worth noting that the nonlocal generation spectrum also reveals lower threshold levels than respective local spectrum and the nonlocal spectrum from the previous case. Thus, the overall generation properties of the considered laser still resemble conventional DFB laser. However, it is worth underlining that spectral position of the Bragg wavelength is significantly deviated with respect to the local generation spectrum, which substantially alters the resulting generation properties.

In the case of TM-polarized waves, spatial dispersion leads to an increase of refractive index, i.e., $n_{TM}\approx \sqrt{\varepsilon_{xx}}$, see Figure 3b, which causes significant redshift of the stop band as well as substantially decreases threshold generation levels with respect to the spectrum predicted with the help of local approach, compare Figure 5d and Figure 6d. Again, the local approach fails to correctly predict generation properties, including the position of Bragg wavelength and the levels of generation thresholds, compare Figure 6b and Figure 6d, which significantly influences the laser operation and, in the case of, e.g., narrow gain bandwidth, may lead to lack of generation.

#### 3.2.4. Case 4–150 nm Dielectric Layer

**Figure 7:**
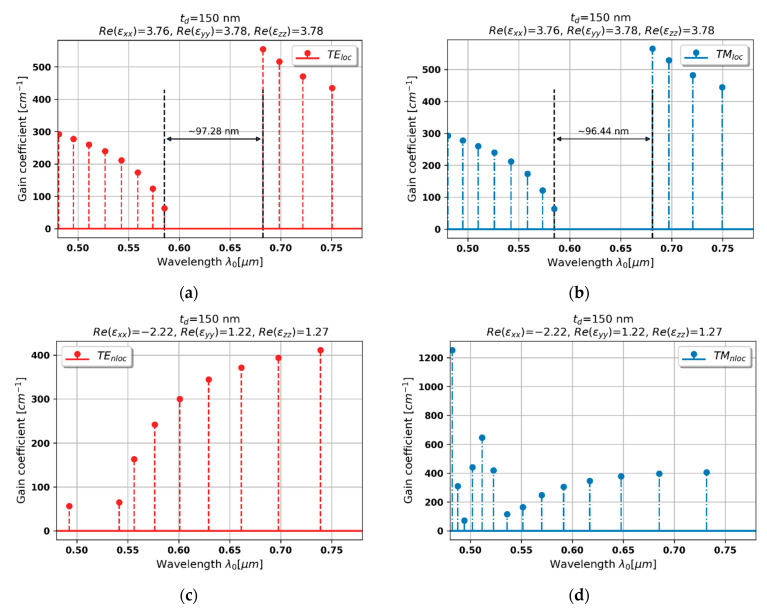
Modal spectra for TE (**a**,**c**) and TM-polarized light (**b**,**d**) for a PHC laser based on hyperbolic medium with $t_{ZnO}$ = 150 nm dielectric layer (black double-headed arrow denotes spectral width of stop band of the spectrum).

In the case of unit cell on 150 nm dielectric layer, the dielectric dispersion is preserved only for TE-polarized waves, while for TM polarization, medium reveals Type II hyperbolic dispersion, see Figure 3a,b. Thus, due to the further decrease of the respective refractive index $n_{TE}\approx \sqrt{\varepsilon_{yy}}$, the stop band for TE-polarized light of nonlocal generation spectrum is again shifted towards shorter wavelengths with respect to the local spectrum, compare Figure 7a and Figure 7c. It is worth noting that, in comparison to previous cases, the levels of generation threshold on both sides of the stop band become symmetrical, see Figure 7c, which is similar to the behavior of a DFB laser with pure index modulation [40].

On the other hand, the behavior of nonlocal generation spectrum for TM-light polarization is considerably altered in comparison with the spectrum predicted with the help of the local response, compare Figure 7b and Figure 7d. It can be observed that there is no observable stop band within the considered spectral range. Such a drastic change is caused by the occurrence of Type II hyperbolic dispersion of HMM structure, see Figure 3b, which leads to dominance of gain/loss coupling in the overall feedback mechanism. Such a spectrum may lead to interesting generation properties, such as high side-mode suppression ratio (~15 dB), for the TM mode of the lowest generation threshold (@494 nm), which may be utilized to obtain single polarization generation.

#### 3.2.5. Case 5–200 nm Dielectric Layer

**Figure 8:**
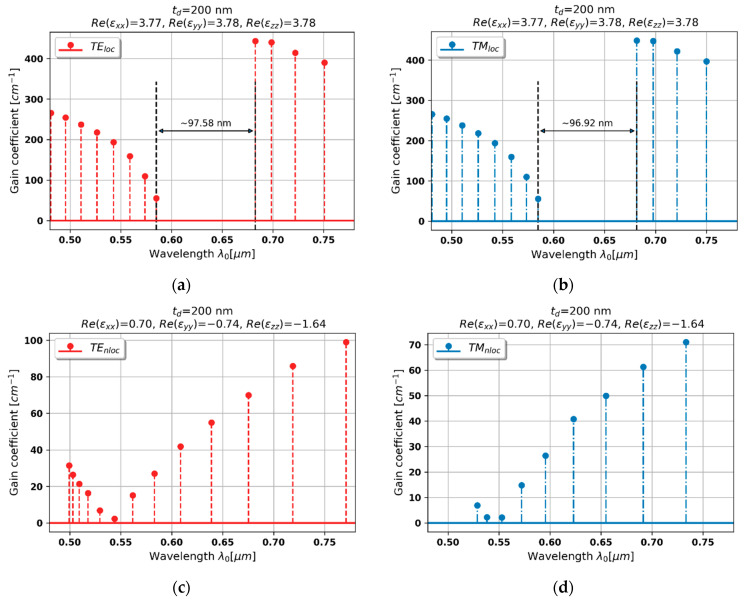
Modal spectra for TE (**a**,**c**) and TM-polarized light (**b**,**d**) for a PHC laser based on hyperbolic medium with $t_{ZnO}$ = 200 nm dielectric layer (black double-headed arrow denotes spectral width of stop band of the spectrum).

By accounting spatial dispersion in hyperbolic medium based on 200 nm dielectric layer, it is possible to predict the metallic dispersion for TE-polarized waves and epsilon-near-zero for TM polarization, see Figure 3a,b. Due to the dominant contribution of loss/gain coupling in distributed feedback, the Bragg wavelengths for both polarizations are blueshifted, compare Figure 8a,b and Figure 8c,d. On the other hand, the overall level of generation threshold is substantially lowered with respect to behavior predicted with the help of local approximation.

Moreover, the modes with the lowest threshold for both polarizations are located around λ = 0.55 μm and spectrally separated by approximately 6 nm (which is equivalent to ~6 THz for the considered spectral range), which may enable simultaneous generation of TE- and TM-polarized waves at different frequencies with use of a single active medium. It is also worth noting that the spectral separation may be controlled with application of appropriate gain medium or adjustment of HMM’s unit cell geometry.

#### 3.2.6. Case 6–250 nm Dielectric Layer

**Figure 9:**
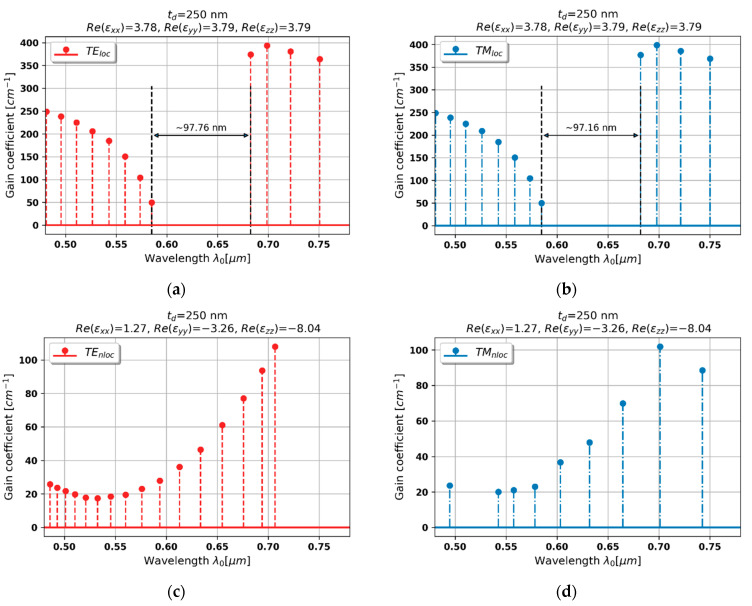
Modal spectra for TE (**a**,**c**) and TM-polarized light (**b**,**d**) for a PHC laser based on hyperbolic medium with $t_{ZnO}$ = 250 nm dielectric layer (black double-headed arrow denotes spectral width of stop band of the spectrum).

The last example is an HMM structure based on 250 nm dielectric layer embedded in a PHC laser. In this case, metallic dispersion is still encountered by TE-polarized waves travelling in the HMM medium, while waves of TM polarization perceive dielectric dispersion gain, see Figure 3a,b. The generation properties predicted with the help of local approximation are very similar to previously observed local generation spectra, compare Figure 9a,b with Figure 8a,b. However, generation spectra for both light polarizations are again substantially altered by accounting for spatial dispersion. In particular, metallic dispersion for TE-polarized waves causes strong gain/loss coupling, which results in a symmetrical spectrum with minimal threshold mode located the center resembling lasing in a DFB with loss/gain modulation, see Figure 9c.

On the other hand, due to the fact that in our analysis we consider only propagation along *z* axis, the Type I hyperbolic dispersion is perceived by TM-polarized waves as conventional dielectric dispersion. Again, lower optical density of dielectric medium leads to blueshift and narrowing of the stop band as well as lowering of the level of generation thresholds, see Figure 9d.

### 3.3. Local and Nonlocal Generation Spectra for a Realistic Gain Curve

In this section, we present local and nonlocal generation spectra of PHC lasers operating in the presence of strong spatial dispersion, which is granted by application of sufficiently thick dielectric layers in the unit cell of hyperbolic medium, i.e., *t_d_* = 150 and 200 nm, that corresponds to the case 4 and 5 of the previous section. Similarly, as in the previous section, the size of the basic cell of a PHC structure is calculated based on local permittivity components. Such an approach allows us to correctly illustrate the behavior which is expected based on the local approach and compare it with more realistic generation properties that are calculated by accounting the influence of spatial dispersion. For this purpose, we also assumed a gain medium with a realistic Lorentzian-like gain curve with central wavelength located at *λ*_c_ = 600 nm, maximal gain *g*_max_ = 80 cm^−1^, and bandwidth *λ*_FWHM_ = 80 nm, which correspond to parameters of polystyrene doped with PM605 dye molecules [42].

**Figure 10:**
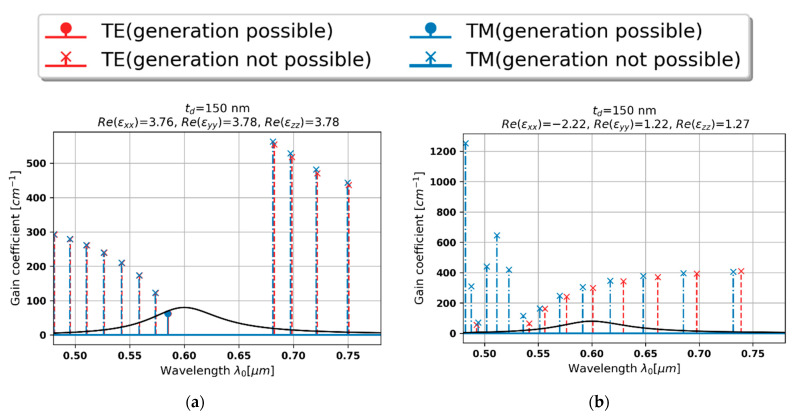

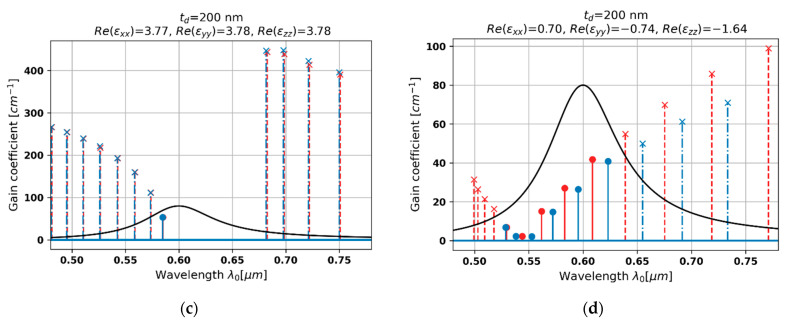
Local (**a**,**c**) and nonlocal generation spectra (**b**,**d**) for PHC lasers based on various dielectric layer thicknesses $t_{d}$ = 150 (**a**,**b**) and 200 nm (**c**,**d**).

In the case of dielectric layer of 150 nm thickness, instead of single mode generation for both polarizations at a single frequency, which is predicted with the help of local approach, the nonlocal generation spectrum indicate that no generation will occur, due to insufficient gain provided by the considered gain medium, see Figure 10a,b.

On the other hand, the second case, i.e., $t_{d}$ = 200 nm, presents significantly diverse behavior, see Figure 10c,d. Instead of single mode generation predicted with the help of local approximation, it is possible to obtain multimode generation for both polarizations. Moreover, since spectra for TE- and TM-polarized waves are shifted in relation to each other, it is possible to obtain simultaneous generation of both polarizations at two distinctively different frequencies (so-called orthogonally polarized beam generation). In this particular case, i.e., for the given PHC structure and gain curve, the most preferred modes are located at 583 and 595 nm for TE and TM polarization, respectively. Thus, the spectral separation between generated modes is 12 nm, which is equivalent to ~10THz and may be considered as useful in many practical applications requiring tunable signal generation, displacement, or force/pressure measurements [52].

## 4. Conclusions

In this study, we have investigated the role of spatial dispersion in shaping threshold lasing in DFB laser based on photonic hypercrystal. For the purpose of analysis, we have employed two well-established methods, namely: effective medium theory for description of hyperbolic medium, as well as transfer matrix method for modeling light propagation and calculating spectra of threshold generation. In the course of our investigation, we have demonstrated that properties of nonlocal generation spectra may significantly vary from behavior predicted with the help of the local approximation. In particular, it has been shown that a nonlocal approach enables correct anticipation of a number of key generation features, including spectral position of Bragg wavelengths or levels of generation thresholds. Moreover, we have presented generation phenomena that arise in the presence of strong nonlocality, such as generation of light of selected light polarization (TE or TM) or simultaneous generation of both TE- and TM-polarized waves at different frequencies (~10 THz separation). We believe that the presented analysis proves that nonlocality is not only crucial to be accounted for in the design process, but may also lead to new phenomena that are not achievable when the spatial dispersion is weak.

## Data Availability

All reported data and tools are available on request.
